# *Drosophila melanogaster* as a model system for studying the effects of porcine rotavirus on intestinal immunity

**DOI:** 10.3389/fcimb.2025.1621846

**Published:** 2025-07-28

**Authors:** Xiao Chun Wang, Shuang Deng, Liyun Yu, Rongan Cao, Liangyu Li

**Affiliations:** ^1^ College of Food Science, Heilongjiang Bayi Agricultural University, Daqing, China; ^2^ National Coarse Cereals Engineering Research Center, Daqing, China; ^3^ College of Life Science and Technology, Heilongjiang Bayi Agricultural University, Daqing, China

**Keywords:** porcine rotavirus (PoRV), pathogen, intestinal immunity, JAK/STAT signaling, *D. melanogaster*

## Abstract

**Introduction:**

*Drosophila melanogaster* is a quintessential model organism that has been used in many scientific studies. The intestinal immune response of flies is a critical component of their innate immune system. Given that flies primarily consume decaying organic matter, harmful microorganisms present in their food can enter the intestine, leading to frequent infections by exogenous pathogens. When these pathogens are introduced into the intestinal environment, a cascade of immune responses is triggered within the intestinal tissue, aimed at preserving the integrity of the intestinal barrier and ensuring the proper physiological functions of the gut. Porcine rotavirus (PoRV) is a key pathogen that causes diarrhea in pigs, and PoRV infection can significantly reduce piglet survival rates.

**Methods:**

In this study, wild-type flies were orally administered PoRV to establish an effective intestinal damage animal model, and a detailed investigation of the antiviral immune defense mechanism in the fly intestine was performed.

**Results and Discussion:**

Our study revealed that PoRV infection caused a reduction in the survival rate of flies and an increase in intestinal epithelial cell death. Concurrently, PoRV infection significantly promoted the proliferation and differentiation of intestinal cells, contributing to the maintenance of intestinal homeostasis. After the activation of JAK/STAT signaling in the intestines of infected *Drosophila*, there was an increase in the levels of reactive oxygen species (ROS). This elevation was concomitant with the release of antimicrobial peptides (AMPs), which play a crucial role in pathogen clearance. Additionally, we identified substantial aggregation of hemocytes in the midgut. The composition of the intestinal microbiota also underwent changes, potentially playing a role in intestinal immune defense. Moreover, PoRV can evade clearance via the RNA interference (RNAi) pathway. In summary, PoRV infection in the fly intestine activates multiple immune defense mechanisms to eliminate the pathogen, offering a theoretical basis for PoRV prevention and control.

## 1 Introduction

*Drosophila melanogaster*, commonly known as the fruit fly, is exposed to a wide variety of pathogens in its natural habitats, including bacteria, viruses, fungi, and parasites, thereby increasing its risk of infection. These pathogens have the potential to adversely affect the health and survival of flies and may also influence their reproductive success and population dynamics by compromising their immune system and physiological functions. Consequently, flies have evolved an intricate immune system that combats these pathogens and ensures their survival. Pathogens can invade the fly body through multiple routes, such as oral ingestion, surface contact, air inhalation, and reproductive transmission (Thakur et al., 2019). Moreover, the gut microbiota has been found to be essential in the fly immune response, impacting its ability to resist pathogens (Raza et al., 2020).

*D. melanogaster* serves as a crucial model organism for investigating immune defense mechanisms following pathogen invasion. Upon entry of a pathogen into the fly gut, a series of intricate immune responses are initiated to protect the host from infection. Initially, pathogen invasion frequently causes cellular damage, leading to the release of endogenous factors such as reactive oxygen species (ROS) (Kosakamoto et al., 2020). ROS not only directly target pathogens but also function as vital signaling molecules that activate immune signaling pathways, including the JNK pathway, thereby facilitating the activation and response of immune cells (Myers et al., 2018). The immune response of the fly relies on the activation of the JAK/STAT signaling pathway. During intestinal infections, JAK/STAT signaling is activated, promoting the regeneration of intestinal epithelial cells and the differentiation of immune cells and thus preserving gut homeostasis (Jiang et al., 2009; Mongelli et al., 2022). Additionally, the expression of antimicrobial peptides (AMPs) in the gut and fat body constitutes a significant mechanism of immune defense in the fly, effectively eliminating invading pathogens (Wu et al., 2012). The Toll and Imd signaling pathways, which represent two principal NF-κB-related pathways in *D. melanogaster*, are activated after pathogen recognition (Nehme et al., 2011; Zhu et al., 2013; Csonka et al., 2021). These pathways induce the expression of immune effector molecules, thereby enhancing the host immune response. Hemocytes, which are capable of phagocytosing pathogens and secreting signaling molecules, are integral to the regulation of the systemic immune response (Tassetto et al., 2017). RNA interference (RNAi) functions as a fundamental antiviral mechanism, inhibiting viral replication through the small interfering RNA (siRNA) pathway (Mussabekova et al., 2017). Finally, the gut microbiota is essential for maintaining immune homeostasis; its dysregulation can result in excessive immune responses and inflammation (Kosakamoto et al., 2020). Investigating these immune defense mechanisms in *D. melanogaster* provides valuable insights into the human immune system and the pathogenesis of diseases. The use of flies as hosts for viral infection research is highly beneficial because their innate immune system has been extensively studied.

Porcine rotavirus (PoRV) is a significant pathogen responsible for acute diarrhea in piglets, leading to elevated morbidity and mortality rates. Group A rotaviruses are identified as the primary etiological agents of rotavirus-associated diarrhea in pigs, affecting them both pre- and postweaning (Crawford et al., 2017). PoRV is a double-stranded RNA virus that encodes six structural proteins, designated VP1, VP2, VP3, VP4, VP6, and VP7. In addition, it encodes six nonstructural proteins, NSP1, NSP2, NSP3, NSP4, NSP5, and NSP6 (Cui et al., 2019). VP6 plays a vital role in polymerase function and the stability of the viral core and is highly antigenic and immunogenic. These characteristics make VP6 useful in diagnostic assays for detecting PoRV (Vlasova et al., 2017). A previous study demonstrated the successful production of double-layered rotavirus-like particles (DVLPs) utilizing a bicistronic expression system in stably transformed *D. melanogaster* S2 cells, with the aim of developing an effective alternative vaccine against rotavirus (Lee et al., 2010). PoRV is a common enterovirus that primarily causes diarrhea and other symptoms by infecting the intestinal epithelial cells of the host. The innate immune system serves as the initial defense against invading pathogens, primarily triggering the immune response by identifying pathogen-associated molecular patterns (PAMPs) (Villena et al., 2016). In porcine intestinal epithelial cells, rotavirus binds to pattern recognition receptors (PRRs), such as RIG-I and MDA5, which are essential for detecting viral RNA and initiating signaling cascades that lead to the production of interferons (IFNs) and other antiviral molecules (Uzri and Greenberg, 2013; Ishizuka et al., 2016). The RIG-I signaling pathway is particularly pivotal in recognizing double-stranded RNA viruses and inhibiting their replication by activating interferon-β (IFN-β) or interferon-λ (IFN-λ) (Zhao et al., 2015; Hou et al., 2025). However, rotavirus can circumvent the host innate immune response by utilizing mechanisms involving the NSP1 and VP3 proteins, which suppress interferon production and signaling, thereby facilitating viral replication and dissemination (Morelli et al., 2015; López et al., 2016). Despite recent research revealing the immune evasion strategy of PoRV, the precise regulatory mechanism of innate immunity remains unclear and requires further investigation.

In this study, oral administration of PoRV reduced the survival rates and regulated the proliferation and differentiation of intestinal cells in *D. melanogaster*. Moreover, PoRV caused oxidative stress and cell death, which aided in pathogen clearance by activating the JAK/STAT pathway, the secretion of AMPs, and the promotion of hemocyte aggregation. Moreover, PoRV was found to evade the immune system via RNAi in flies. These findings provide a basis for further studies on the mechanisms by which PoRV affects intestinal immune responses.

## 2 Materials and methods

### 2.1 Fly stocks and cultures

The wild-type *W^1118^*, *esg^ts^-Gal4;UAS-GFP*, *NP1-Gal4/CyO*, *10XSTAT-GFP*, and *hml-Gal4;2XEGFP* fly stocks were kindly gifted by Professor Li Hua Jin from Northeast Forestry University. Flies were cultured in a constant-temperature incubator at 25°C with a relative humidity of 60% on a 12-h light/dark cycle.

### 2.2 Culturing PoRV in Vero E6 cells

To culture porcine rotavirus (PoRV) in Vero E6 cells, the following streamlined protocol was used. Vero E6 cells were seeded in a T25 flask at a density of 1 × 10^5^ cells/mL. The mixture was incubated at 37°C with 5% CO_2_ for 24 h until 80-90% confluence was reached. A 10-fold serial dilution of the PoRV stock mixture in DMEM (Gibco, USA) without fetal bovine serum (FBS, Gibco, USA) was prepared. The culture medium was removed, and 1 mL of diluted virus was added to the flask. The mixture was incubated for an hour at 37°C to allow virus adsorption. The medium was replaced with 5 mL of DMEM containing 10% FBS, 1% penicillin-streptomycin and 1% L-glutamine (Thermo Fisher Scientific, USA), and the infected cells were incubated for 4-6 days and monitored daily for cytopathic effects (CPEs), such as cell rounding and detachment. When significant CPEs were observed, the supernatant was harvested. The mixture was centrifuged at 3,000 rpm for 10 min to remove cell debris. The clarified supernatant was aliquoted and stored at −80°C. The virus was titrated via tissue culture infectious dose 50 (TCID50) assays. The TCID50 was calculated using the Reed-Muench method on the basis of the dilution at which 50% of the wells presented CPEs, and the resulting TCID50 of PoRV was 10^−4.25^/0.1 mL. Vero E6 cells and the PoRV strain were obtained from R. Zhao (Zhou et al., 2023).

### 2.3 Oral administration

To conduct the feeding experiments, adult flies aged three to five days were used. These experiments were conducted in vials, each containing 15 male and 15 female flies. After a fasting period of two hours in an empty vial, the flies were transferred to a vial containing five layers of filter paper saturated with a 5% sucrose solution (w/v) with or without PoRV at 100 TCID50, 200 TCID50, and 300 TCID50. The filter papers were replaced daily, and the number of surviving flies was recorded at each transfer over a period of 10 or 15 days. Each experiment was independently conducted at least three times.

### 2.4 Immunostaining

The intestines extracted from 15-20 male flies were fixed in 4% paraformaldehyde at room temperature for 25 min to prepare them for immunostaining. The samples were subsequently incubated in a blocking solution consisting of 0.1% Tween 20 and 5% normal goat serum in phosphate-buffered saline (PBS) for one hour at room temperature. The samples were then incubated with primary antibodies overnight at 4°C, followed by incubation with secondary antibodies according to standard protocols. The samples were ultimately mounted in Slow Fade Diamond Antifade Mountant (Thermo Fisher Scientific, USA) and analyzed using a Leica Microsystems microscope. The primary antibodies used were rabbit anti-phospho-H3 (pH 3, 1:200; Abcam, UK) and rabbit anti-GFP (1:200; Thermo Fisher Scientific, USA). Secondary antibodies conjugated with Alexa Fluor 488 (Thermo Fisher Scientific, USA) were used at a 1:200 dilution. The samples were subsequently stained with DAPI (1:500, Thermo Fisher Scientific, USA) for 5 min and mounted with 90% glycerol diluted in PBS. Each experiment was independently conducted at least three times.

### 2.5 7-AAD assay

Dead cells were detected using 7-aminoactinomycin D (7-AAD, Thermo Fisher Scientific, USA). The gut imaging and staining procedures followed previously established protocols (Li et al., 2013). Briefly, 12-15 adult male intestines were dissected in cold PBS and incubated with 7-AAD at a concentration of 5 µg/mL in PBS for 30 min at room temperature in the dark. The samples were then washed three times with PBS. For immunostaining, the dissected male fly intestines were fixed in 4% paraformaldehyde for 30 min at room temperature. Each experiment was independently conducted at least three times.

### 2.6 Determination of ROS levels

The intestines of 10-12 male flies were incubated with dihydroethidium (DHE, 5 μM, Thermo Fisher Scientific, USA) at ambient temperature for 30 min, followed by fixation in 4% formaldehyde for 10 min. Subsequently, the samples were stained with DAPI for 5 min and mounted with 90% glycerol (diluted in PBS). The posterior midgut was then examined using a Leica Microsystems microscope. This experimental procedure was independently replicated a minimum of three times.

### 2.7 Quantitative polymerase chain reaction

Adult male flies were exposed to PoRV (300 TCID50) for 72 h. Following the established protocol, total RNA was isolated from 30-40 intestines or 20 adult males using TRIzol reagent (Invitrogen, USA). qPCR was performed using a Bio-Rad CFX96Touch (Bio-Rad, UAS) with the BeyoFast™ SYBR Green One-Step qRT-PCR Kit (Beyotime Biotech, China). Each sample was analyzed in triplicate. The primers, listed in **Supplementary Table S1**, were supplied by Beyotime (Beyotime Biotech Co., Ltd., China). The Ct (threshold cycle) values were used to quantify target gene expression relative to a reference gene. The 2^(−ΔΔCt) method was used to calculate the fold changes.

### 2.8 Effect of PoRV on the intestinal microbial community

Adult males were infected with PoRV (300 TCID50) for 72 h, and 90-100 intestines were dissected for 16S rDNA microbial community analysis. This detection was carried out by Shanghai Majorbio Biotechnology Co., Ltd. An analysis was conducted on the alpha and beta diversity of the intestinal flora, along with a comparison of the relative abundance of the top 15 genera.

### 2.9 Statistical analysis

Images of the midgut of the fruit fly intestine were obtained using a Leica Microsystems microscope. All the numerical data, including the intensity of the gut and the number of cells, were analyzed using ImageJ software. Two-tailed unpaired Student’s t tests were conducted using Prism software (GraphPad 9.5.0) for statistical analysis. The results were considered statistically significant at *P*<0.05; ****, ***, ** and * indicate *P*<0.0001, *P*<0.001, *P*<0.01 and *P*<0.05, respectively; and ns indicates no significant difference. In the graphs, the error bars represent the standard error of the means. All the quantifications were performed in a blinded manner.

## 3 Results

### 3.1 PoRV decreases survival rates *in vivo*

**Figure 1:**
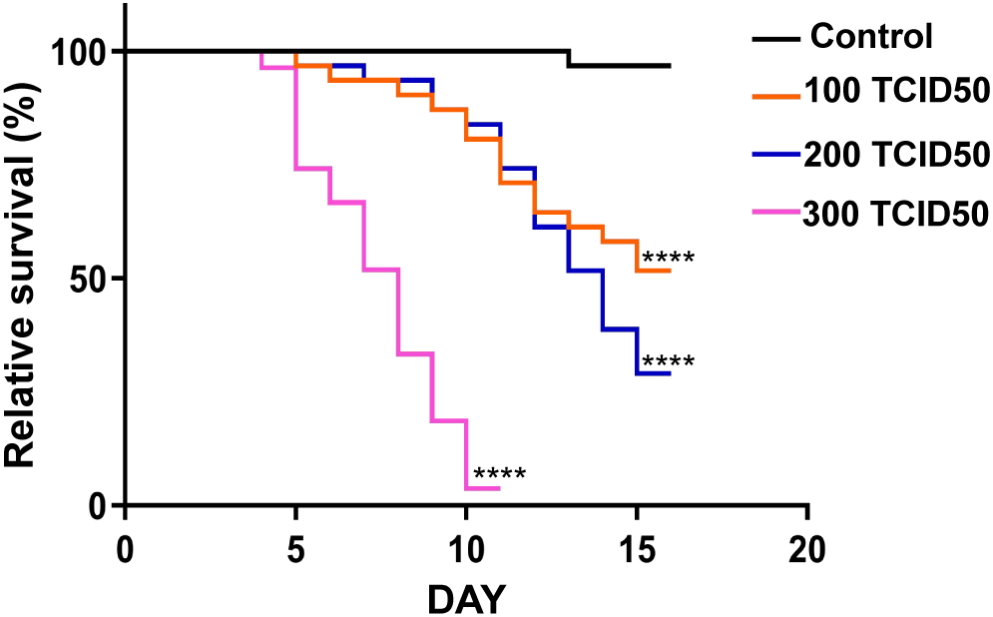
Survival rates of the control and experimental groups of *Drosophila. W^1118^* adult flies were cultured in standard medium or medium supplemented with PoRV. These experiments were conducted in vials, each containing 15 male and 15 female flies. Control, sucrose (5%, w/v); experimental groups, RoRV (5% sucrose plus the 100 TCID50, 200 TCID50 or 300 TCID50 values of RoRV). At least three replicates were performed for each treatment. Survival differences were analyzed via the log-rank test. ****P <0.0001 vs. the control group.

The model organism *D. melanogaster* has a clear genetic background and rapid reproduction rate and is easy to manipulate. It is often used to study the infection mechanisms of mammalian viruses and host immune responses. Although flies and mammals have undergone significant evolutionary divergence, they still share many signaling pathways and genes related to immunity. Viruses, such as *Drosophila* C virus (DCV), Nora virus, and Muthill virus, can greatly decrease the survival rates of flies after infection (Kutzer et al., 2023; Wallace and Obbard, 2024). To investigate the pathogenicity of PoRV in *D. melanogaster*, the flies were orally administered a standard diet with or without PoRV at 100 TCID50, 200 TCID50 or 300TCID50. As shown in **Figure 1**, the survival rates of the flies decreased with increasing PoRV concentration. We found that the survival rates of the flies administered PoRV at 100 TCID50 and 200 TCID50 decreased by 50% and 73.34%, respectively, within 15 days, whereas the survival rate of the flies administered PoRV at 300 TCID50 decreased by 86.67% within 10 days. This result not only indicates the pathogenicity of PoRV to flies but also shows that the pathogenicity is proportional to the viral dose.

### 3.2 PoRV induced the proliferation of intestinal cells

**Figure 2:**
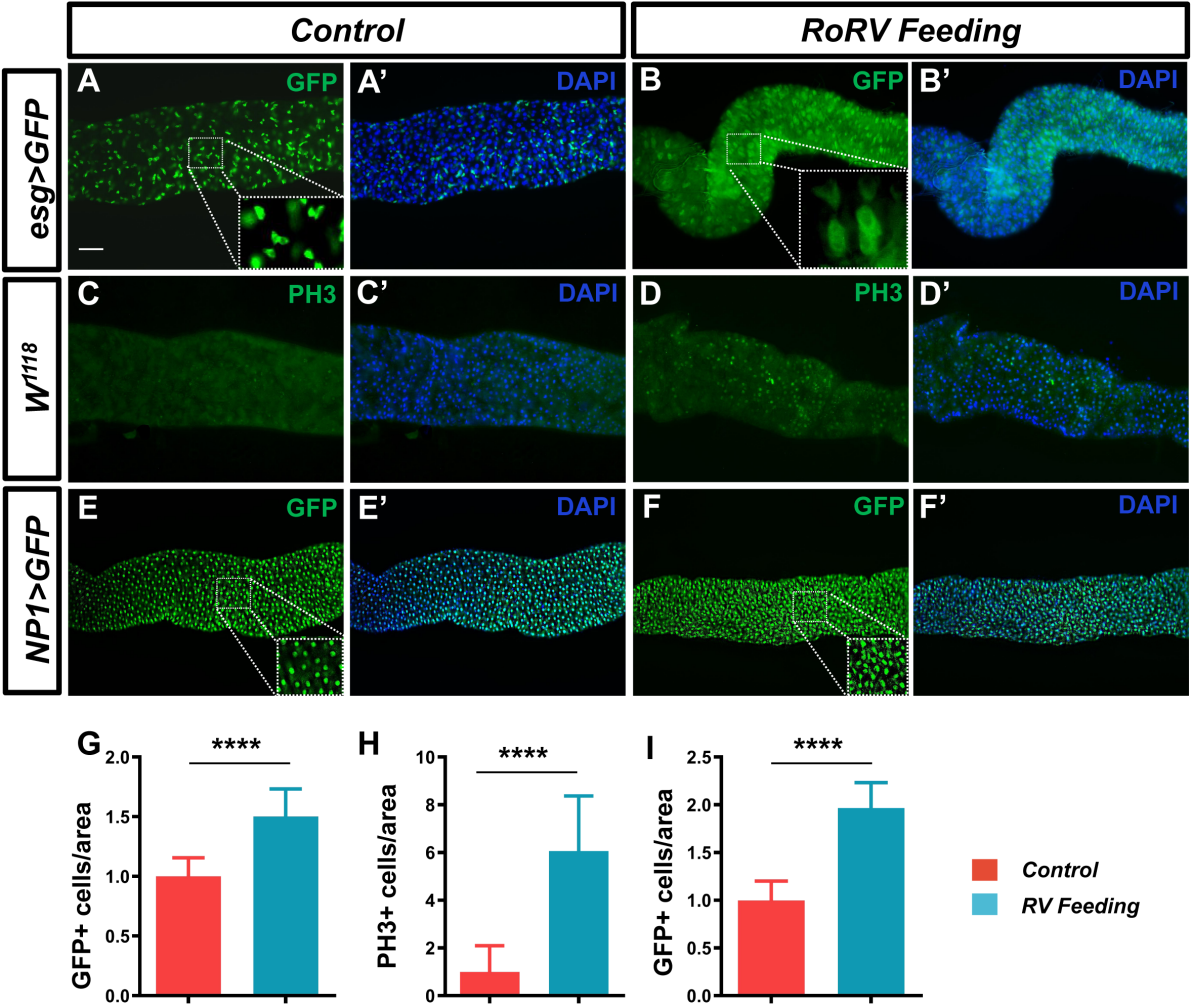
PoRV treatment changes the number of intestinal cells in the *Drosophila* gut. Three- to five-day-old *esg>GFP, W^1118^* or *NP1>GFP* adult flies were fed sucrose or 300 TCID50 of RoRV plus 5% sucrose for 72 h. **(A, A’, B, B’)** Progenitor cells were stained with anti-GFP antibodies (green). **(C, C’, D, D’)** The proliferation of ISCs in the posterior midgut was evaluated with anti-PH3 antibodies (green). **(E, E’, F, F’)** EC cells were stained with anti-GFP antibodies (green). DAPI, nuclei (blue). **(H)** Quantification of the number of PH3+ cells per unit area of the midgut in **C** and **D**, n > 15. **(G, I)** The number of GFP+ cells per unit area of the midgut in **A, B** and **E, F** are shown, n>15. Scale bars: 50 μm.

After pathogens enter the fly intestine, they activate a variety of defense mechanisms in the gut. These mechanisms help to resist the invasion of pathogens and maintain intestinal homeostasis. When pathogens invade the intestines, intestinal stem cells (ISCs) will replace damaged cells through increased compensatory proliferation (Amcheslavsky et al., 2009). This cell regeneration mechanism helps to maintain the integrity of the intestinal epithelium and prevent further invasion by pathogens. Therefore, we further investigated the effects of PoRV on intestinal cell homeostasis. We used *esg-Gal4^ts^;UAS-GFP* to specifically label ISCs and EBs in the fly midgut following treatment with PoRV at 300 TCID50 for 72 h. PoRV infection increased the number of GFP-positive cells by 50.32% compared with that in the control groups (**Figures 2A, A’, B, B’, G**). These findings indicated that PoRV feeding promoted the differentiation of ISCs and EBs. Interestingly, after PoRV feeding, the GFP-positive cells were larger and had a faint GFP signal, which probably indicated that they were premature ECs (preECs) (Lei et al., 2022).

Upon infection, in adult tissues and organs, resident stem cells are triggered to maintain homeostasis. Consequently, we employed an anti-PH3 antibody to stain isolated *D. melanogaster* guts, allowing us to identify the mitotic phase of ISCs. Our findings indicated that the PoRV-treated group presented a significant increase in the number of PH3-positive cells in the gut, which was 6-fold greater than that in the control group (**Figures 2C, C’, D, D’, H**). To determine the impact of PoRV on EC proliferation, *NP1-Gal4;UAS-GFP* was used to label ECs with GFP. The number of ECs was significantly increased by approximately 96.59% in PoRV-infected flies compared with control flies after anti-GFP antibody staining (**Figures 2E, E’, F, F’, I**). These results indicate that PoRV significantly promotes the excessive growth of intestinal cells in flies and activates intestinal immunity to maintain intestinal homeostasis.

### 3.3 PoRV induced intestinal epithelial cell death and oxidative stress

**Figure 3:**
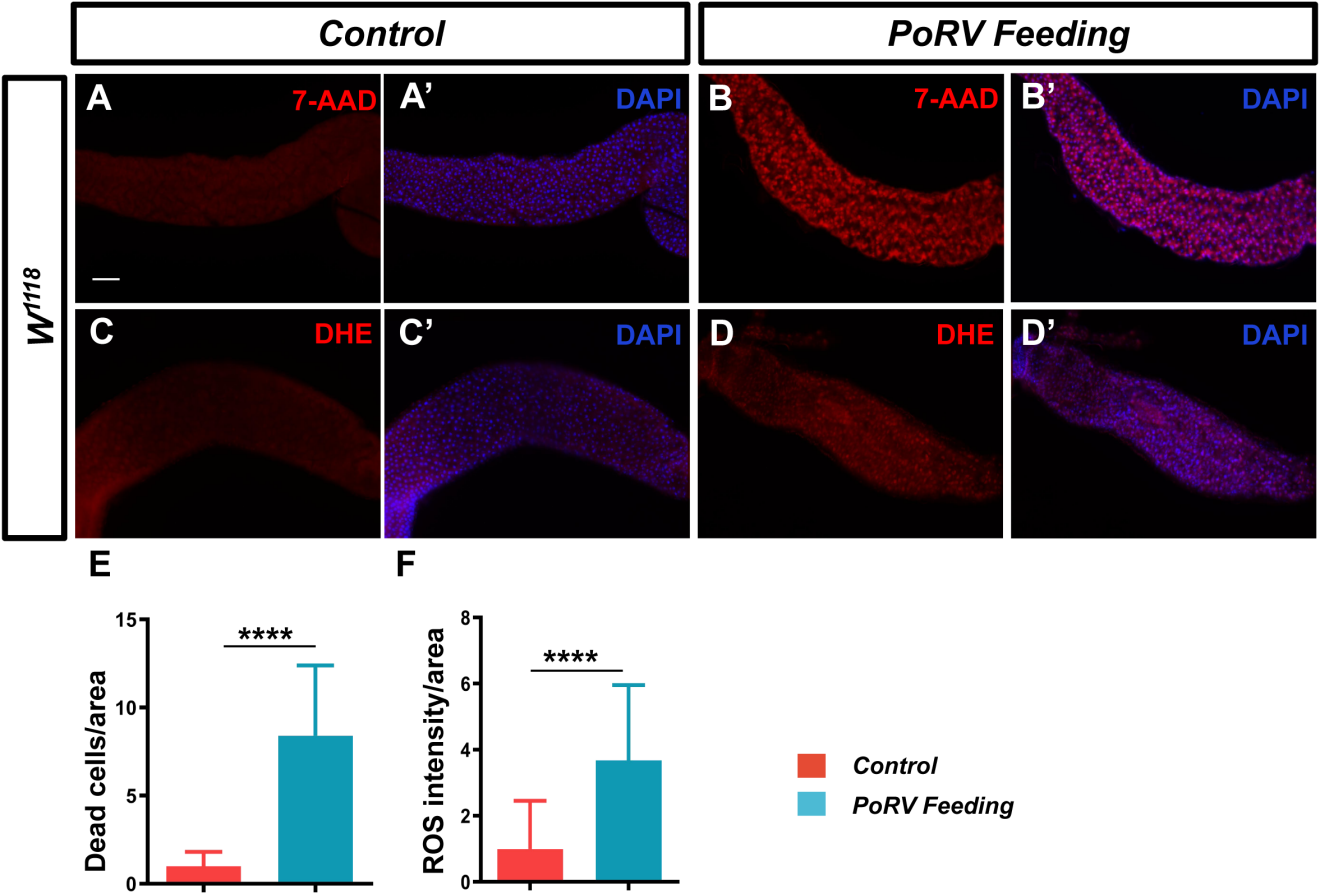
PoRV induced cell death and increased ROS levels. Three- to five-day-old *W^1118^* adult flies were fed 5% sucrose or 300 TCID50 of RoRV plus 5% sucrose for 72 h. **(A, A’, B, B’)** Dead cells were detected with 7-AAD. **(C, C’, D, D’)** ROS levels in the adult fly midgut were evaluated via DHE staining. **(E)** Quantification of the number of dead cells per unit area of the midgut in **A** and **B**, n > 12. **(F)** Quantification of DHE intensity per unit area of the midgut in **C** and **D**, n > 10. Scale bars: 50 μm.

The analysis of the aforementioned content suggests that a decreased survival rate and excessive proliferation and differentiation of intestinal cells may result from damage to the intestinal mucosal barrier induced by pathogen invasion, which subsequently disrupts the normal physiological functions of the intestine. Therefore, adult flies were treated with PoRV at 300 TCID50 for 72 h, and the guts were isolated for 7-AAD staining. Compared with the control group, the PoRV-infected group presented a greater quantity of dead epithelial cells, as expected (**Figures 3A, A’, B, B’, E**).

When pathogens invade, the host organism frequently initiates an oxidative stress response. In the fly intestine, a large amount of ROS is produced in response to pathogen invasion (Amcheslavsky et al., 2009). ROS function as the primary defense against infections by pathogens in the intestines. Using a DHE staining assay, we measured the ROS levels in the midgut. We found that flies infected with PoRV had a significant increase in ROS fluorescence intensity (**Figures 3C, C’, D, D’, F**). These results indicate that PoRV can increase the levels of ROS, leading to a redox imbalance in the host and compromising the integrity of intestinal epithelial cells.

### 3.4 PoRV activated the JAK/STAT pathway

**Figure 4:**
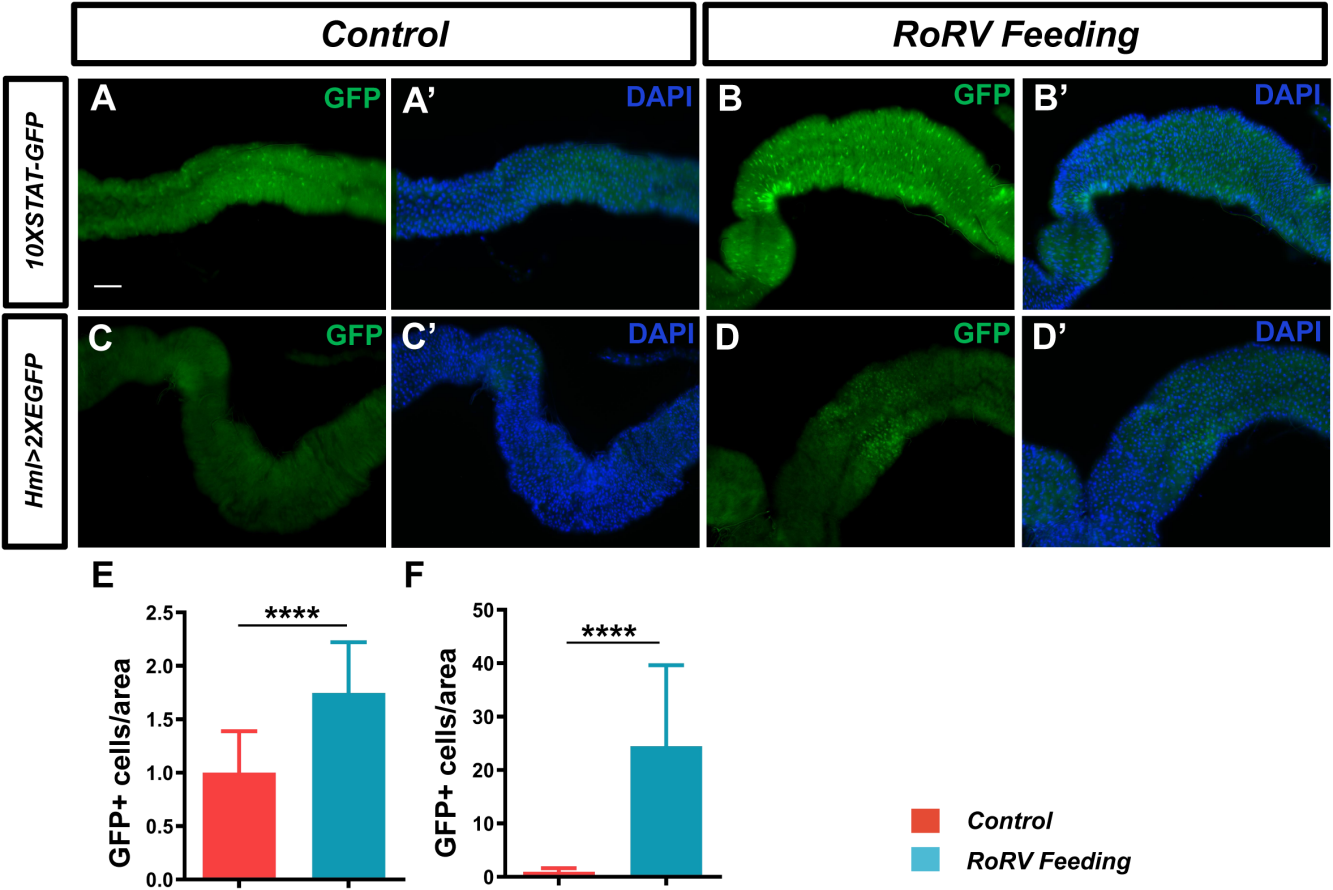
PoRV feeding activated the JAK/STAT pathway and increased midgut hemocyte numbers. **(A, A’, B, B’)** JAK/STAT pathway activity was assessed using the 10XSTAT-GFP reporter. The number of GFP+ cells was greater in the flies infected with PoRV than in the uninfected flies. **(C, C’, D, D’)** High ROS levels mediated by PoRV led to the aggregation of hemocytes. A *hml>2XEGFP* transgene was used to label hemocytes. **(E, F)** The numbers of GFP+ cells per unit area of the midgut in **A, B** and **C, D** are shown, n > 15. Scale bars: 50 μm. ****P < 0.0001, scale bars: 50 μm.

The JAK/STAT pathway is a critical signaling pathway involved in various cellular processes, including immune function, cell growth, differentiation, and cancer progression. Following viral infection, JAK/STAT pathway activation is essential for antiviral defense in flies. Studies have shown that after infection with DCV, the JAK/STAT pathway is activated, thereby enhancing the immune response to the virus (Zhu et al., 2013). Specifically, the activation of the JAK/STAT pathway can induce the expression of STAT-responsive factors, which improve resistance to viral infection in flies (Huang et al., 2023). We employed *10XSTAT-GFP* transgenic flies to observe the activation of the JAK/STAT signaling pathway, where GFP indicates the target gene *Socs36E* of this pathway. Notably, the GFP level increased by approximately 74.71% after PoRV injury compared with that of the control (**Figures 4A, A’, B, B’, E**). These results show that activating the JAK/STAT pathway enhances gut immunity against intestinal damage caused by PoRV infection.

### 3.5 Increased midgut hemocyte numbers in flies infected with PoRV

Following the invasion of the pathogen into the gut of the fly, the aggregation of hemocytes plays a critical role in the immune response of an organism (Madi et al., 2021). The immune system in *D. melanogaster* offers an essential framework for elucidating the immune mechanisms present in more complex organisms. The presence of localized necrotic cells can initiate a systemic immune response (Kosakamoto et al., 2020). To determine whether PoRV treatment affects the number of hemocytes in the midgut, the *hml>2XEGFP* fly line was used to label hemocytes. After 72 h of feeding, the PoRV treatment group presented a greater number of GFP-positive cells in the midgut than did the control group (**Figures 4C, C’, D, D’, F**). This finding indicates that PoRV infection in the fly intestine results in intestinal damage and necrosis, subsequently triggering a systemic immune response to eliminate the virus from the organism.

### 3.6 Oral administration of PoRV resulted in excessive AMP levels and Imd pathway activation

**Figure 5:**
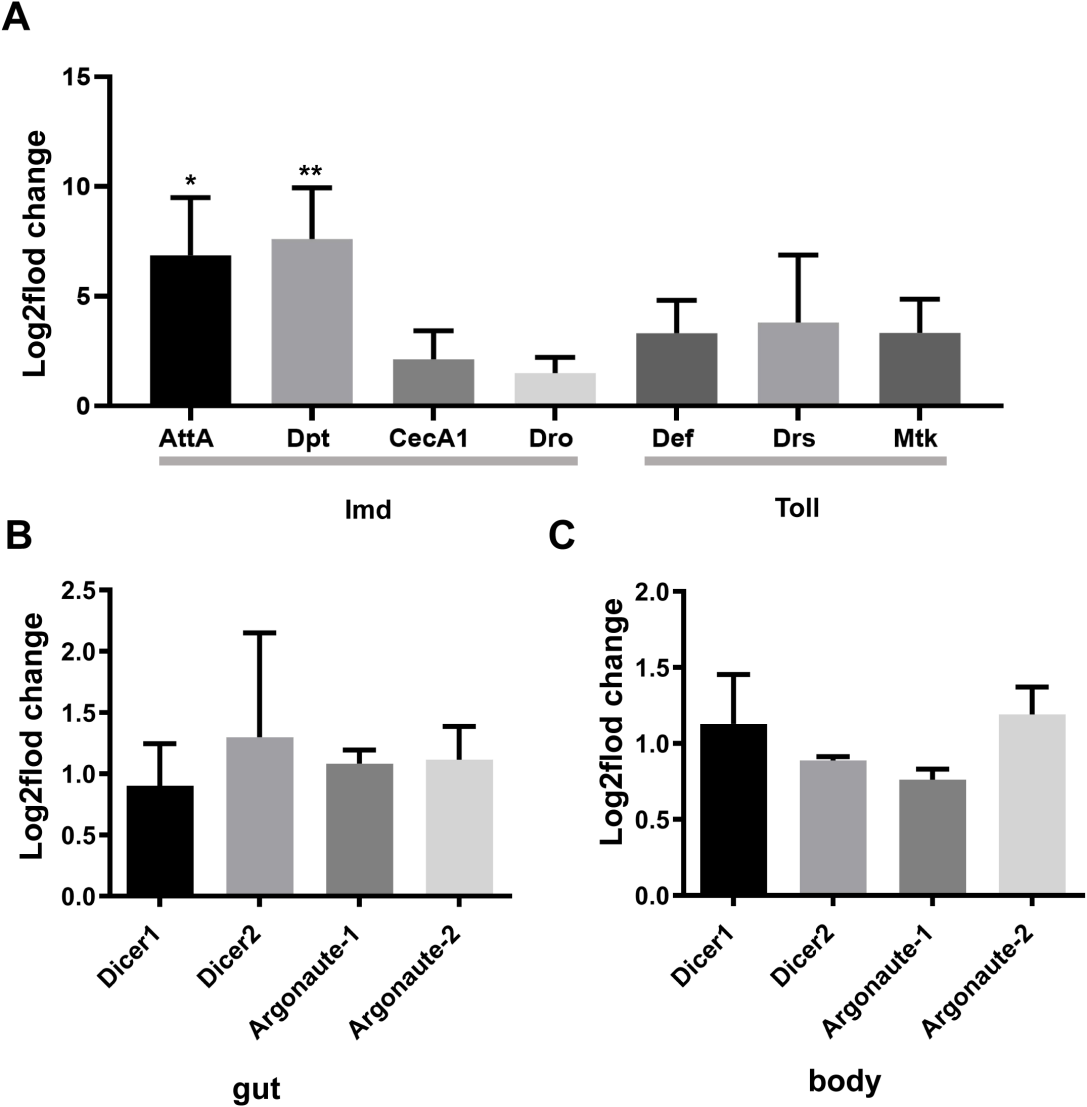
qRT-PCR analysis of antimicrobial peptides (AMPs) and siRNA/miRNA pathway member levels in the adult gut or body. *W^1118^* adult male flies that were treated with 300 TCID50 and their guts were analyzed. **(A)** Relative gene expression of AMPs in the gut. **(B, C)** Relative expression of genes associated with the siRNA/miRNA pathways in the gut and body. Similar expression patterns were observed in three independent experiments. AttA, Attacin A; Dpt, Diptericin; CecA1, Cecropin A1; Dro, Drsosocin A; Def, Defensin; Drs, Drosomycin; Mtk, Metchnikowin.

The innate immune system of *D. melanogaster* includes both cellular and humoral immunity, which cooperate to offer a powerful defense against various pathogens, including bacteria, viruses, fungi, and parasites. In flies, the Toll and Imd signaling pathways are essential for the immune response, particularly in combating pathogen invasion. These two signaling pathways activate a series of signal transduction and amplification processes, ultimately leading to the secretion of AMPs, effectively clearing pathogens from the body (Hwang et al., 2013). Therefore, we explored whether oral administration of PoRV could cause excessive accumulation of AMPs via qRT-PCR. Compared with the uninfected group, the group of flies exposed to PoRV presented 6.9- and 7.6-fold increases in the transcript levels of *AttA* and *Dpt*, respectively (**Figure 5A**). Activation of the Imd signaling pathway, which plays a key role in immune defense against bacteria and viruses, leads to the secretion of the antimicrobial peptides AttA and Dpt by the organism. The lack of the Imd pathway in flies has been shown to increase their vulnerability to viral infections and substantially increase the viral load (Costa et al., 2009). These results indicate that humoral immunity is also involved in the clearance mechanism of PoRV, which is achieved through the secretion of relevant AMPs via the Imd signaling pathway.

### 3.7 PoRV evades the RNAi pathway in flies

RNA interference plays a significant role in antiviral defense in flies by degrading viral RNA, thereby inhibiting the replication and spread of viruses. Research has shown that flies can effectively resist viral infections through RNAi pathways, including the miRNA and siRNA pathways (Sabin et al., 2009). Thus, we analyzed the transcript levels of key genes in the RNAi pathway to assess their involvement in the antiviral immune response. Interestingly, after PoRV administration for 72 h, there were no obvious changes in the intestine or in the entire adult body (**Figures 5B, C**). These findings indicated that PoRV evaded the RNAi pathway in flies.

### 3.8 The impact of PoRV on the composition of the intestinal microbiota

**Figure 6:**
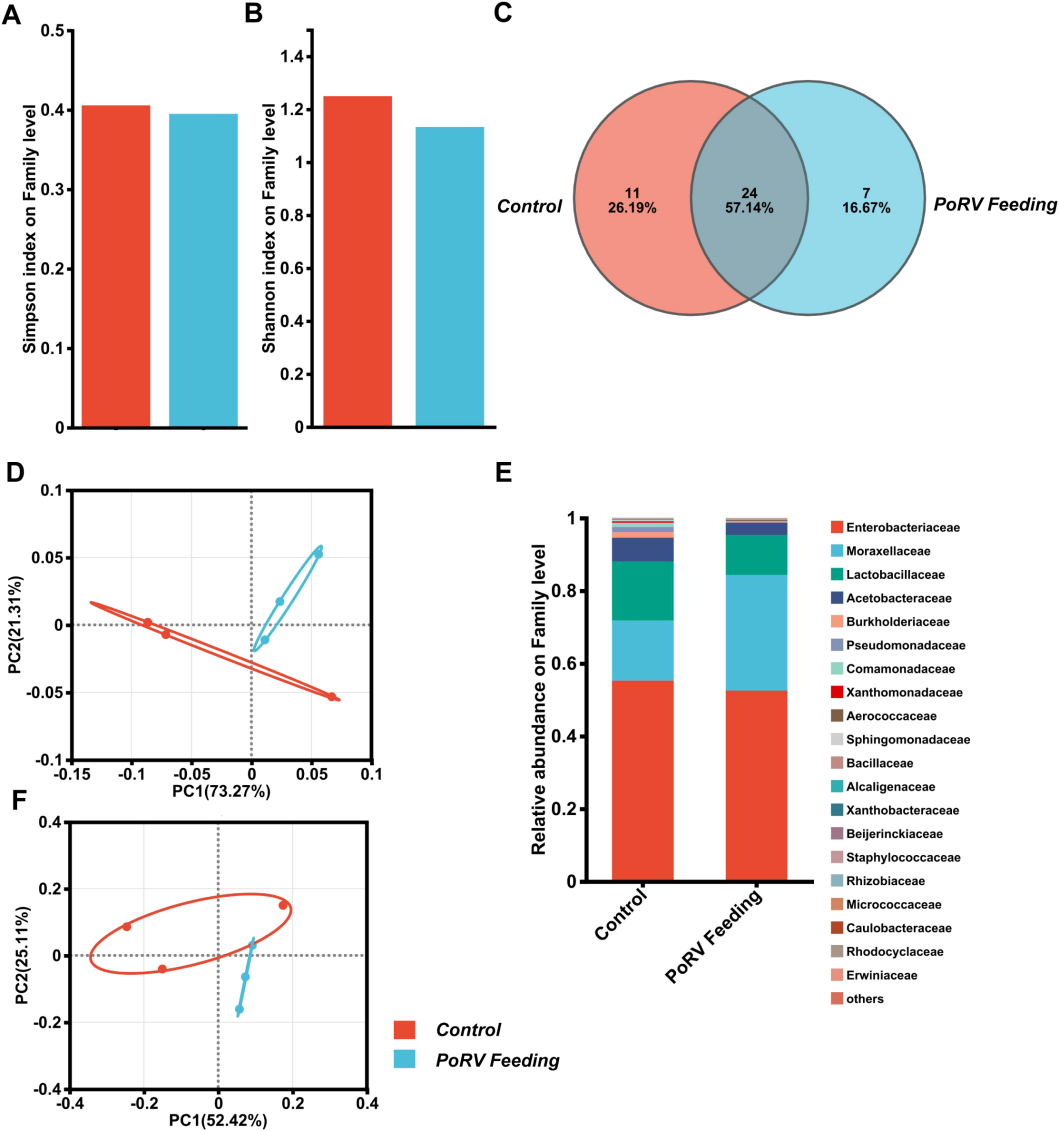
PoRV infection affects the gut microbiota composition. **(A, B)** α diversity. The bar plot analysis shows the biodiversity values for the Simpson and Shannon indices. No statistically relevant differences were observed. **(C)** Venn diagram showing the number of families unique to and shared among different groups. **(D, F)** Principal coordinate analysis (PCoA) for both UniFrac distances. (Student’s t test: weighted *P* = 0.386; unweighted *P* = 0.406). **(E)** Bacterial composition of the control and PoRV groups. Relative taxonomic abundances are shown at the family level. All bacterial taxa present at < 1% relative abundance were grouped into the “Other” classification.

The gut microbiota of *D. melanogaster* is vital for resisting viral infections. Studies have revealed that it strengthens the host’s immune defenses through different mechanisms, limiting the replication and spread of viruses (Sansone et al., 2015; Yang et al., 2021). We explored how intestinal infections affect the composition of the gut microbiota in flies using 16S rRNA sequencing. We conducted an oral infection of 5-day-old conventional wild-type flies, which had native microbiota, treated with PoRV at 300 TCID50 and dissected their guts 72 h later for 16S rRNA examination. According to the Simpson and Shannon indices, 16S rRNA amplicon sequencing revealed no significant difference in intestinal bacterial community α diversity between uninfected controls and PoRV-infected flies (**Figures 6A, B**; **Supplementary Figures S1A-F**). Furthermore, β diversity analyses were used to examine the similarities between microbial communities across all the samples. According to permutation-based Student’s t tests, there was no significant difference between the samples (**Figures 6D, F**). Moreover, a Venn diagram analysis revealed that 35 and 31 bacterial families were differentially abundant between the control and PoRV-infected groups, respectively. Moreover, the numbers of unique families were 11 and 7, respectively (**Figure 6C**). We subsequently assessed the relative abundance of the key families in the 16S sequencing data. We found that infection led to a reduction in the abundance of the *Enterobacteriaceae*, *Lactobacillaceae* and *Acetobacteraceae* families (**Figure 6E**; **Supplementary Figures S1G, H**). Taken together, while there were compositional changes after infection, the microbial α- and β-diversity indices showed no significant differences between the control and PoRV treatment groups.

## 4 Discussion

The immune system of *D. melanogaster*, while structurally and functionally distinct from that of more complex animals, remains highly evolutionarily conserved, making *D. melanogaster* an important model animal for immune mechanism research. Moreover, *D. melanogaster* lacks an adaptive immune system and relies on innate immunity to combat pathogens, making it an ideal subject for innate immunity research. This characteristic renders flies invaluable for elucidating the fundamental mechanisms of the immune system. For example, extensive research on the role of Toll-like receptors in antifungal immunity has not only deepened our understanding of innate immune responses but also facilitated the identification of Toll-like receptors in mammals, thereby significantly advancing our comprehension of the immune system (Rämet, 2012). Moreover, studies have demonstrated that the Imd signaling pathway is pivotal in regulating the expression of AMPs, which are essential for the proper immune function of flies (Myllymäki et al., 2014). Furthermore, the immune system of flies involves complex induction responses and restriction factors, which collectively contribute to the control of viral infections (Mussabekova et al., 2017). Research on the immune system of *D. melanogaster* has extended beyond antimicrobial and antiviral defense mechanisms to encompass investigations into host-pathogen interactions. Given the genetic and signaling pathway similarities between *D. melanogaster* and mammals, *D. melanogaster* is extensively utilized to elucidate novel mechanisms of infection and disease progression (Younes et al., 2020). Additionally, *D. melanogaster* is used often in immune research to explore the concept of immune memory. Although *D. melanogaster* lacks a classical adaptive immune system, its innate immune system may exhibit certain memory-like characteristics, suggesting a novel perspective on the evolution of the immune system (Arch et al., 2022). Furthermore, the immune system of *D. melanogaster* is intricately linked to other physiological systems. Studies have demonstrated that the immune response in *D. melanogaster* is regulated by, and in turn influences, endocrine and metabolic signaling systems, thereby establishing a feedback loop that maintains physiological homeostasis (Buchon et al., 2014). This investigation into systemic regulation provides significant insights into the coordination of the immune system at the organismal level. The regulation of the gut microbiome is considered a crucial factor in combating insulin resistance symptoms. Research has shown that *Acetobacter* and *Lactobacillus* can alleviate insulin resistance symptoms in fruit flies by blocking the JNK-JAK/STAT pathway. This suggests that probiotics supplementation and modulation of the JNK-JAK/STAT pathway activity may have potential therapeutic effects in diabetes control (Meng et al., 2024). Consequently, *D. melanogaster* was chosen as the experimental animal model for PoRV infection, as it is more favorable for studying the innate immune response.

**Figure 7:**
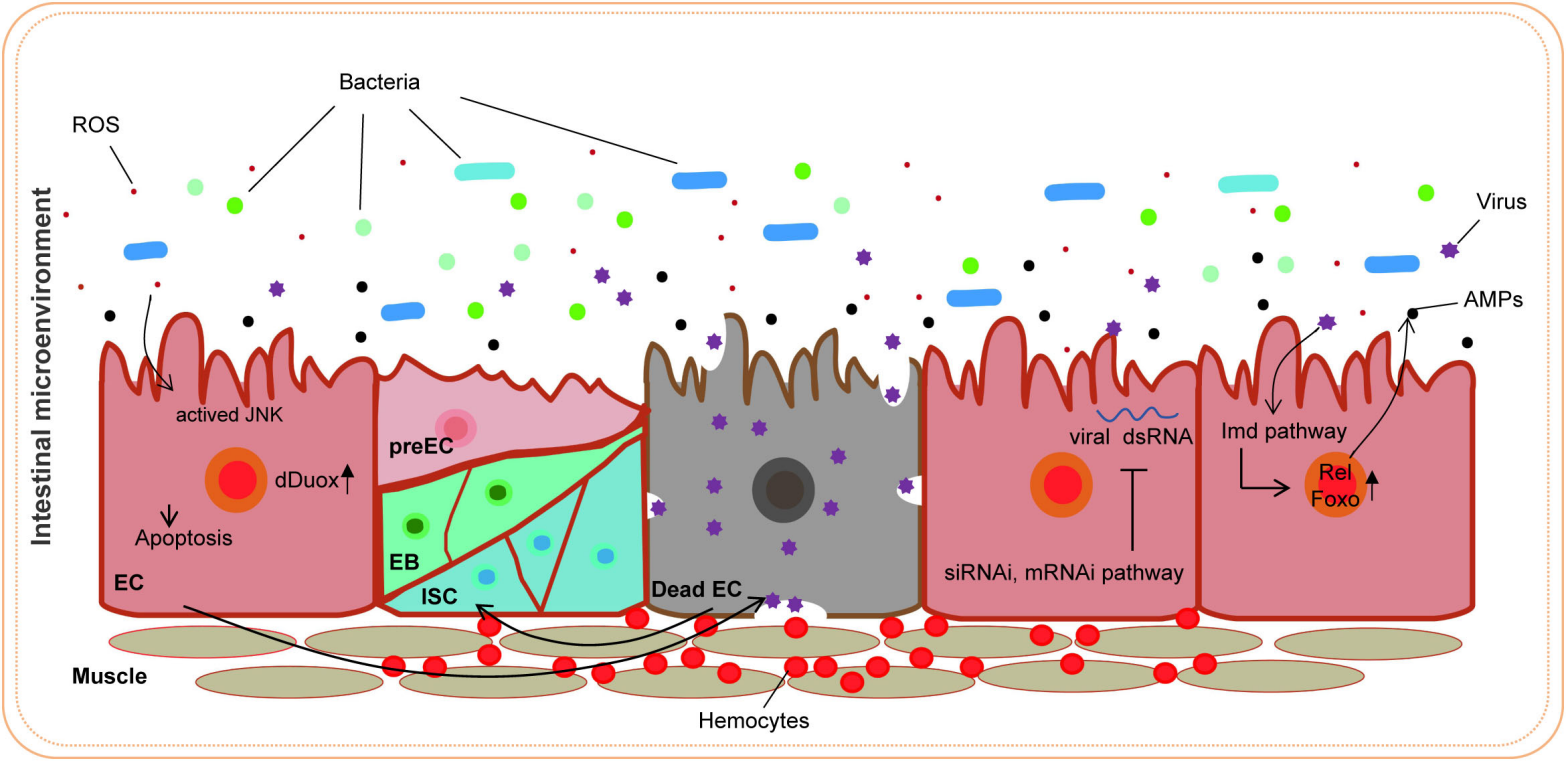
Antiviral strategies in the *D. melanogaster* intestine.

Research advances have led to an increased understanding of the antiviral mechanism of *D. melanogaster*. Following a viral infection, multiple antiviral pathways are activated and work synergistically to eliminate pathogens in the fly. Pathways associated with apoptosis, the oxidative stress response, hemocyte aggregation, and antimicrobial peptide secretion and the JAK/STAT pathway are involved in this response to viral infection (**Figure 7**). Our findings indicate that PoRV infection in flies leads to intestinal damage, which subsequently reduces fly survival rates and results in the death of epithelial cells (**Figures 1**, **3A, B**). Research has indicated that fly survival rates decrease significantly following infection with DCV. This reduction is caused by the virus replicating and spreading throughout the body. Viral infection stimulates the immune system of flies, leading to various physiological changes (Chtarbanova et al., 2014; Gupta et al., 2017). The influence of viral infections on the intestinal cells of *D. melanogaster* represents a complex and significant domain of research. Studies have demonstrated that viral infections can disrupt intestinal homeostasis through a variety of mechanisms. When intestinal homeostasis is compromised, excessive proliferation and differentiation of intestinal stem cells can occur to compensate for the loss of intestinal cells. This abnormal proliferation of intestinal stem cells is a primary contributor to the reduced lifespan observed in fruit flies (Koehler et al., 2017). Viruses exhibit specific tropism when infecting the intestinal cells of fruit flies, which triggers the activation of distinct pathways to maintain homeostasis within the intestinal tissues. For example, *Drosophila* A virus (DAV) initially targets ECs and is occasionally detected in ISCs or EEs (Nigg et al., 2024). DAV infection induces persistent ISC proliferation through the activation of epidermal growth factor receptor (EGFR) and JNK signaling in intestinal cells, but Sting-dependent NF-κB (Relish) activation is required for this effect. This results in developmental abnormalities of the intestine, compromised intestinal barrier function, and a shortened lifespan. An alternative scenario involves the infection of fruit flies by the Nora virus, which initially targets ISCs and activates the JAK/STAT signaling pathway (Franchet et al., 2025). This activation promotes ISC proliferation, aiding fruit flies in mitigating infection-induced damage. However, such proliferation is not invariably advantageous, as excessive ISC proliferation can result in abnormal intestinal tissue growth, negatively impacting overall health and reducing the lifespan of fruit flies. Our findings indicate that abnormal ISC proliferation in the intestines of fruit flies is accompanied by a significant increase in JAK/STAT signaling. Consequently, we hypothesize that PoRV likely initially infects ISCs and utilizes JAK/STAT signaling to promote proliferation, thereby maintaining intestinal tissue integrity. Future research will focus on elucidating the mechanisms by which PoRV induces excessive ISC proliferation. Moreover, ROS are essential in the antiviral immune response of flies. Studies have demonstrated that virus infection causes a significant increase in ROS levels in flies. Increased ROS levels can alter cell function by inducing apoptosis and altering signaling pathway activity (West and Silverman, 2018; Liang et al., 2024). We also detected a significant increase in ROS levels in the PoRV-infected fly intestines (**Figures 3C, D**). In addition, the activation of the JAK/STAT signaling pathway, which is important for regulating immune responses and increasing fly survival rates (Merkling et al., 2015), is related to the production of ROS. These findings indicate that JAK/STAT signaling pathway activation in the fly intestine infected with PoRV could be related to intestinal cell damage and oxidative stress. Notably, the Imd signaling pathway is integral to resistance against viral infections in flies. Research indicates that flock house virus (FHV) infection in flies triggers the activation of the Imd signaling pathway, subsequently resulting in the secretion of AttA and Dpt AMPs, which facilitate the clearance of the viruses (Shen et al., 2022). Similarly, the intestines of the flies infected with PoRV presented markedly increased mRNA levels of AttA and Dpt (**Figure 5A**). Taken together, when *D. melanogaster* is infected with PoRV, it triggers a range of immune defense mechanisms within the organism to eliminate the pathogen.

However, although the RNAi pathway is believed to be involved in combating viruses in *D. melanogaster*, the results revealed that there was no differential regulation of the pertinent genes in the entire adult fly or its intestine (**Figures 5B, C**). During a viral infection, the RNA interference (RNAi) pathway in *D. melanogaster* is generally activated as a defense mechanism against viral invasion. Nonetheless, there are instances where the RNAi pathway remains unactivated postinfection, potentially due to viral suppression strategies. For example, the B2 protein of FHV serves as an RNAi inhibitor, effectively obstructing the activation of the RNAi pathway and thereby facilitating viral replication and dissemination within the host (Han et al., 2011). Furthermore, the RNAi pathway in flies is subject to regulation by additional factors. Research has demonstrated that the FOXO transcription factor can modulate the expression of key genes within the RNAi pathway. During viral infection, FOXO activity is increased, which enhances the efficiency of RNAi. These findings suggest that flies can adapt to varying environmental and metabolic conditions by modulating FOXO activity, thereby optimizing gene silencing efficiency (Spellberg and Marr, 2015). Studies have shown that PoRV can escape the host-mediated RNA interference (RNAi) immune response through a variety of mechanisms to successfully replicate and spread in the host. PoRV achieves biphasic regulation of RNAi by modulating the degradation of Argonaute2 (AGO2). In the early stages of infection, PoRV triggers the degradation of AGO2 through its NSP1, thereby inhibiting the siRNA-mediated RNAi response. However, in the later stages of infection, RNAi function is restored, indicating that the virus-mediated regulation of RNAi is time-dependent (Mukhopadhyay et al., 2019). The study also revealed how PoRV manipulates the host cell’s signaling pathways by encoding virus-like small RNA, such as RV-vsRNA1755. By targeting the insulin-like growth factor 1 receptor (IGF1R), RV-vsRNA1755 blocks the PI3K/Akt pathway, triggering autophagy but ultimately inhibiting its maturation. This mechanism provides favorable conditions for rotavirus to survive and replicate within the host cell (Zhou et al., 2018). Thus, we hypothesized that PoRV infection in *D. melanogaster* activated an immune evasion mechanism involving the RNAi pathway, which merits further investigation and exploration.

The gut microbiota of *D. melanogaster* plays an essential role in the physiological and immune functions of the host. Research indicates that the symbiotic bacterial community within the fly gut undergoes substantial alterations during viral infections, potentially impacting the nutrient metabolism and immune response of the host (Haider et al., 2025). The dominant bacterial genera in the fly gut are *Acetobacter* and *Lactobacillus* (Wong et al., 2011; Meng et al., 2024). These bacteria are integral to the nutrient metabolism of flies, particularly in the assimilation and metabolism of amino acids and other nutrients (Yamada et al., 2015). Moreover, certain marine prebiotics, such as agar oligosaccharides (AOS), have been found to improve intestinal inflammation in fruit flies by regulating gut microbiota, immune gene expression, and autophagy. This regulation not only increases the α-diversity of the gut microbiota but also reduces the abundance of bacteria that are prone to causing infections, such as *Klebsiella aerogenes*, thereby extending the lifespan of fruit flies (Ma et al., 2021). However, viral infections in flies can lead to changes in the abundance of these dominant bacterial genera, thereby influencing the nutrient metabolic capacity of the host (Sommer and Newell, 2019). Although the α-diversity and β-diversity of the intestinal flora were not significantly different after the infection of PoRV flies, the abundances of *Enterobacteriaceae*, *Lactobacillaceae* and *Acetobacteraceae* decreased (**Figure 6**). These results further indicate that the intestinal commensal flora may contribute to resistance against PoRV infection.

## 5 Conclusion

PoRV is a major pathogen responsible for severe diarrhea in piglets. Understanding its immune mechanisms is essential for creating effective vaccines and treatments. Our investigation into the antiviral immune mechanisms of *D. melanogaster* infected with PoRV revealed that the coordinated action of multiple immune defense strategies is needed to eliminate the virus in the gut. Furthermore, the intestinal microbiota may also play a role in this process, although the virus can inhibit the RNAi pathway. Therefore, a thorough investigation into the immunological mechanisms and evasion strategies of rotavirus aids in comprehending its pathophysiology and offers a crucial scientific foundation for creating more effective vaccines and therapies.

## Data Availability

The raw data supporting the conclusions of this article will be made available by the authors, without undue reservation.
