# Supplementary material for: Drosophila melanogaster as a model system for studying the effects of porcine rotavirus on intestinal immunity
Source: Front Cell Infect Microbiol. 2025 Jul 28;15:1621846. doi: 10.3389/fcimb.2025.1621846 (PMC12336259; doi:10.3389/fcimb.2025.1621846)
Supplement: Supplementary file 1 [file DataSheet1.docx]

Supplementary Material

# Supplementary Figures and Tables

## Supplementary Tables

Table S1 Primers for qPCR.

| **Target gene** | **Primer** |
| --- | --- |
| Defencin (Def) | F: CGCTTTTGCTCTGCTTGCTTGC  R: TAGGTCGCATGTGGCTCGCTTC |
| Drosomycin (Drs) | F:CTTGTTCGCCCTCTTCGCTGTC  R: AGCACTTCAGACTGGGGCTGCA |
| Attacin A (AttA) | F: AGGTTCCTTAACCTCCAATC  R: CATGACCAGCATTGTTGTAG |
| Diptericin (Dpt) | F: ATGCAGTTCACCATTGCCGTC  R: TCCAGCTCGGTTCTGAGTT |
| Cecropin A1 (CecA1) | F: TCTTCGTTTTCGTCGCTCTC  R: CTTGTTGAGCGATTCCCAGT |
| Drosocin (Dro) | F: TGAAGTTCACCATCGTTTTCCTG  R: CACCCATGGCAAAAACGC |
| Metchnikowin (Mtk) | F: GCATCAATCAATTCCCGCCACC  R; CGGCCTCGTATCGAAAATGGG |
| Dicer1 | F: CATTGCGTTCACCTCCAAG  R: TACTGCCGCTCGTTAGCATT |
| Dicer2 | F:GTATGGCGATAGTGTGACTGCGAC  R:GCAGCTTGTTCCGCAGCAATATAGC |
| Argonaute-1 | F: GCGAGGTTTGGTTCGGTTTC;  R: CGTTGATGTCGCGAATGTCC |
| Argonaute-2 | F: CCGGAAGTGACTGTGACAGATCG  R: CCTCCACGCACTGCATTGCTCG |
| PoRV | F: TCTAGAATGGAGGTTCTGTACTCATTG  R: AGCTTCATCATCATCATCATCATTCACTTA ATCAACATGCTTCTA |
| RpL32 | F: AGTCGGATCGATATGCTAAGCTGT  R: TAACCGATGTTGGGCATCAGATACT |

## Supplementary Figures


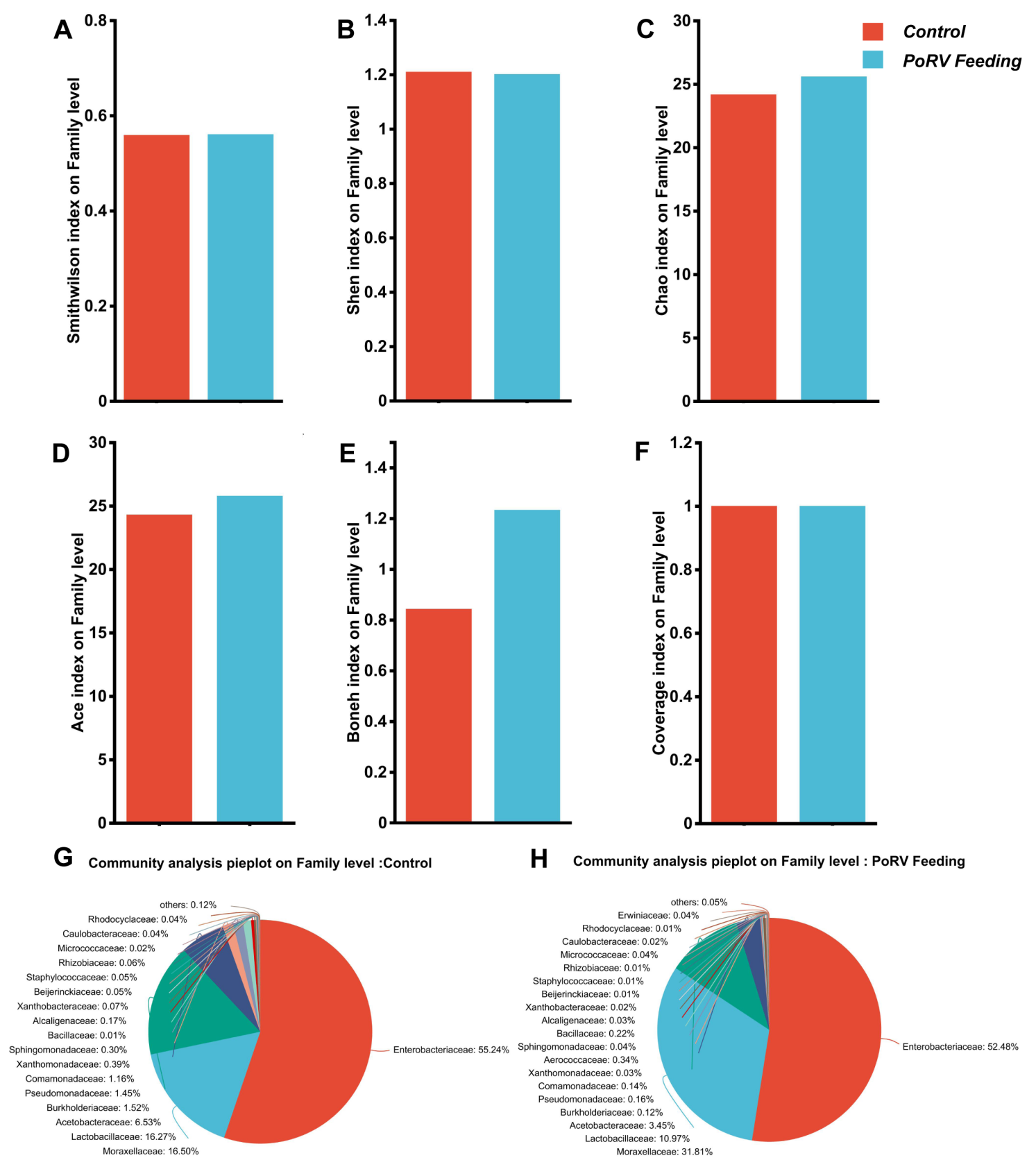


**Supplementary Figure 1.** PoRV feeding files gut microbiota α-diversity and community analysis.


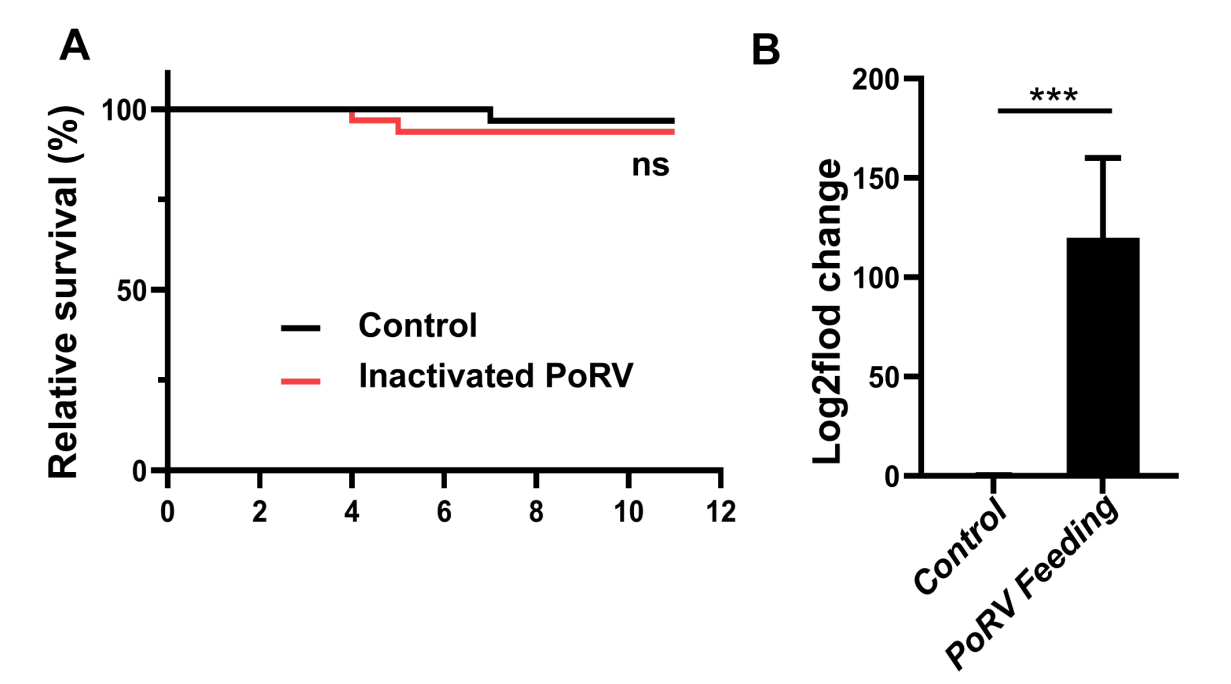


**Supplementary Figure 2.** (A) Survival rates of the control and inactivated PoRV groups of *Drosophila*. At least three replicates were performed for each treatment. Survival differences were analyzed via the log-rank test. ns vs. the control group. (B) qRT–PCR analysis of PoRV RNA levels in the adult gut. Adult male flies that were treated with 300 TCID50 for 72 h, and their guts were analyzed. The intestines extracted from 30–40 male flies were washed in PBS (RNase free) 3–5 times to prepare them for qRT–PCR. ****P* <0.001 vs. the control group.
